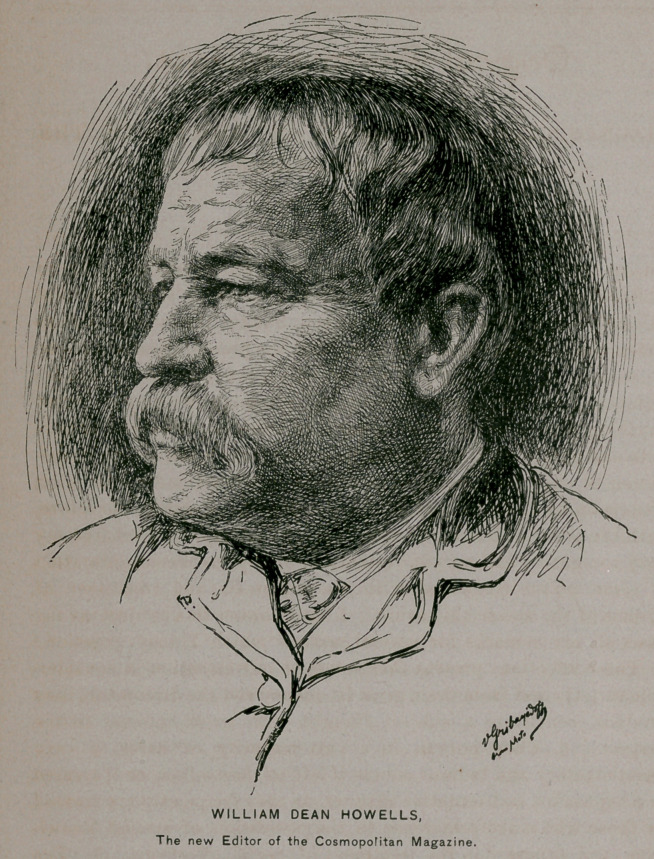# Diagnosis and Treatment of Abscess of the Ano-Rectal Region1Read before the Buffalo Medical and Surgical Association.

**Published:** 1892-06

**Authors:** Edward Clark

**Affiliations:** Lecturer on Diseases of the Rectum in the Medical Department of the University of Buffalo; 271 Franklin Street


Buffalo Medical and Surgical Journal

VOL. XXXI. JUNE, 1892. No. 11.

### Original Communications.

### DIAGNOSIS AND TREATMENT OF ABSCESS OF THE ANO-RECTAL REGION.[1]

BY EDWARD CLARK, M. D.,

Lecturer on Diseases of the Rectum in the Medical Department of the University of Buffalo.

I HAVE accepted the courteous invitation of our presiding officer to read a short paper tonight, not because I hope to say much that will prove of very great interest to you, but for the reason that, as a member of this association, I feel that the presentation of a paper of some sort occasionally is a duty which we owe to our fellow-members and to the association ; and although we may not offer anything new or original, we should, at least, be willing to contribute our mite toward the discharge of our duty, even if our contribution be only a review of some subject intended merely to elicit discussion by the members of our association. I desire, therefore, with the latter object in view, to make an attempt to discharge my duty tonight by calling your attention, briefly, to the consideration of some points connected with the diagnosis and treatment of abscess of the ano-rectal region. I have chosen this subject as the basis of my remarks for several reasons which I may mention : 1. These affections present themselves to our attention in considerable variety, and from their great frequency and the discomfort they produce, constitute a subject fraught with great interest to the surgeon. 2. They pursue, in a vast majority of cases, a very unsatisfactory and tedious course if left to themselves, or if treated in a haphazard and tentative manner, as they frequently are treated by those who make pretension to the possession of special knowledge and fitness for the treatment of rectal affections. 3. The recognition and management of these affections fall properly within a special field of practice, viz., rectal surgery, to the study and cultivation of which I have for a long time exclusively devoted my

1. Read before the Buffalo Medical and Surgical Association.

attention. I have alluded to the fact that abscess in the ano-rectal region presents a number of varieties; hence we find the text-books speaking of marginal abscess, fecal or rectal abscess, more properly known as ischio-rectal abscess and pelvi-rectal abscess. French writers were the first to describe marginal abscess as a distinct variety. This variety is very common, and we most frequently find them resulting from inflamed external hemorrhoids, in which case they are liable to be followed by a slight form of fistula. This form of abscess, however, originates in many ways other than in inflamed hemorrhoids. It sometimes begins as a small furuncle, or boil, in one of the mucous follicles at the margin of the anus. Suppuration of slight cracks or fissures near the anal margin may give rise to it. I have known it to occur from ignorantly injecting an external hemorrhoid with substances intended to effect a cure. Finally, it may be caused by traumatism, or exposure to wet and cold. This form of abscess is generally small and circumscribed, occurring more frequently in men than in women or children. It generally runs a rapid course, and is of no great consequence, except in tubercular or syphilitic patients, in whom it is sometimes followed by marginal ulceration of the anus, which sometimes requires much time and patience to bring up to a perfect cure. When these abscesses are in the formative stage, they produce a good deal of pain and discomfort, owing to the high degree of sensibility which is always present in the region in which these little tumors occur, leading the patient to think that he is suffering from an affection of great gravity. Early and free incision relieves this pain, and this, of course, is the proper treatment.

Ischio-rectal abscess, or the second variety found occurring in the ano-rectal region, is an affection of great importance, for upon its prompt recognition and proper management rest very largely the preventive treatment of that disagreeable malady, fistula-in-ano.

This variety of abscess takes its origin in the loose web of connective tissue and fat surrounding the lower end of the rectum, filling in what is known as the ischio-rectal fossa. This favorite locality for abscess presents all the facilities for the development and rapid spread of purulent collections. And when we take into consideration its anatomical structure and location, we can readily understand why an abscess in this situation attains such an enormous size before external fluctuation manifests itself, or even before the pent-up matter finds its way into the rectum.

I shall not weary you by going into the etiology of these cases, as you are all familiar, no doubt, with the many causes of these troublesome abscesses. The point I wish to emphasize is, that early diagnosis and proper management in these cases are of far greater importance than many of our profession seem to imagine. I am irresistibly inclined to make this statement for the reason that during the past year I have treated no less than five cases of severe fistula resulting from ischio-rectal abscess, which result, according to the clinical history in nearly all these cases, could probably have been avoided if the management of them had fallen into proper hands. I think I am quite safe in asserting that fully fifty per cent. of the fistulæ that we meet with following this form of abscess, could be prevented by early diagnosis and early and energetic management. If this statement is true, it will be readily seen that the treatment of ischio-rectal abscess is a subject well worthy our best thought and attention. When a patient comes to us presenting the symptoms of ischio-rectal abscess, we should lose no time in making an early diagnosis if such condition exists. In order to do this, rectal exploration is absolutely necessary, for, by this means, we can recognize in many cases the presence of deep-seated suppuration in the ischio-rectal fossa long before external fluctuation manifests itself. He who waits for external fluctuation until the patient's buttock becomes brawny, red, and tender, until rigors, fever, etc., manifest themselves, he who waits, I repeat, for these things before resorting to rectal exploration, shows an undue want of familiarity with the proper handling of these cases, and his delay in taking decisive steps to arrest the progress of the affection may give rise to extensive destruction of pelvic tissue, which may cause putrid absorption, involving danger to life. If we are not successful in our first rectal examination, we should repeat our explorations at short intervals, for it is only by timely recognition of the presence of pus that we can hope to succeed with our preventive treatment, which is the great desideratum in the management of these cases. To show the deleterious results of delay, and a masterly inactivity, or perhaps I should say ignorance, in cases of the affection under consideration, I wish to refer briefly to the clinical history of the five cases which I have alluded to above. These patients all consulted me for treatment for fistulæ of recent formation following abscess, and, as my note-book reveals the fact that the cases were all very similar, I will say that the history given by each patient was like unto the following:

A few weeks or months ago, had pain in vicinity of the rectum ; at first slight and indistinct, then gradually growing more severe, especially during the act of defecation. Soon there appeared a slight hardness or induration in the buttock close to the anal aperture. Pain increased in intensity, and began to assume a throbbing character, and was soon so severe that standing, walking, or sitting were quite painful. Was compelled to take to the bed and call in a physician, who gave medicine to relieve pain, and applied tincture of iodine locally to "scatter" (?) the swelling. Pain constantly increasing, of a heavy, throbbing character, accompanied by fever and restlessness. As swelling could not be scattered, poultices were applied, but no use made of the knife. The patient, in answer to a request that the abscess be opened, was told that if any cutting were done a fistula would surely result! After ten or twelve days of intense suffering, the abscess finally broke. Weeks have come and gone and the abscess cavity does not heal, but continues to discharge a purulent matter, which creates a vast amount of local irritation.

It would, perhaps, seem that the above picture is overdrawn, but it is not, and the fact that in one year five cases of ischio-rectal abscess were so grossly mismanaged by men in this city who are doing a large practice, would seem to indicate an absolute necessity for even the much ridiculed specialist in rectal diseases. But to go back to first principles: Given an ischio-rectal abscess, how shall we treat it? The plan that I follow in these cases is as follows: Give the patient a brisk cathartic, and when it has fully operated, wash out the rectum thoroughly with repeated hot enemata. Administer an anesthetic, and when your patient is fully under its influence forcibly stretch and paralyze the sphincter ani muscle. This step of the procedure is very important and absolutely essential to success in these cases, for, in no other way can we secure perfect rest for the parts after operation. It is well known that the alternate contraction and relaxation of the sphincter muscle is a great hindrance to the ready healing of abscesses in this particular locality. Now, take a long, sharp bistoury and lay open the abscess cavity freely, cutting always through the integument outside of the sphincter muscles, the line of incision running at right angles to the nearest point of the anal margin. Introduce the finger or a scoop into the abscess cavity and thoroughly clean it out, breaking down all bands or pockets so that we have one cavity only. Now, syringe the cavity freely with a hot antiseptic solution, put in a drainage tube, or fill the abscess cavity loosely with a long strip of iodoform gauze, allowing it to hang out of the incision to keep the wound open and secure thorough drainage. The cavity should be cleaned daily, and the drainage tube shortened from time to time as the healing proceeds. Keep the patient in bed on a nourishing diet, and in a week or ten days he will be entirely well with no resulting fistula. In speaking of treatment, some authors recommend that the abscess be opened from the rectum. I am of the opinion that this is a process not to be recommended, for we then convert the abscess into a blind internal fistula for the time being, and as such it is likely to remain for a long time, for blind internal fistulæ are more difficult of treatment than either of the other varieties. It will probably be urged by some that this is a very heroic plan of treatment for so slight an affection as ischio-rectal abscess. They may say that it is only necessary in these cases to lay open the abscess and let the patient go about his business, and that it is arrant nonsense to anesthetize a patient and stretch his sphincter ani muscle for such a trifling condition. In answer to such criticism, I would say that experience has shown that ischio-rectal abscess is by no means a trifling affair. It is well known that it frequently leads to severe forms of fistula which may leave the patient a miserable invalid for years, and the plan of treatment I have outlined is our only safeguard against such disastrous results. Opening the abscess freely and scooping it out, as recommended by many, is not sufficient. I am satisfied that stretching the sphincter muscle is the most important step in the management of this affection, for it is only by resorting to this procedure that we can put the parts absolutely at rest, which must in all cases be done in order to prevent the formation of fistulæ and sinuses. That this radical treatment is a necessity is very forcibly brought home to my mind by the cases above alluded to, and more particularly by a case which came under my observation a short time ago, photographs of which I was able to obtain, and which I take pleasure in showing you tonight. The patient, a very intelligent and well-to-do young man, about twenty-five years of age, had an ischio-rectal abscess a few years before I saw him, which was treated on the expectant plan by one of those physicians who thought lightly of the young man's affliction. He poulticed and painted, and waited, and finally after nearly two weeks of intense suffering, the abscess ruptured spontaneously. The patient's physician assured him that now his troubles were over. He allowed him to get up and go about. After discharging freely for a few days, the abscess cavity apparently closed up. In a short time it filled and was allowed to break again. Things went on in this way for a long time, until the young man's buttocks were fairly riddled with sinuses and he became weak and emaciated,—in fact, a veritable invalid. About this time he placed himself under the care of a noted surgeon in New York, where he was at the time, who tried to bring about a radical cure. The ravages of the disease had formed so many fistulous tracks which required opening up, that the result of the operation was disastrous. Here was a strong young man left with permanent incontinence, owing absolutely to the neglect which was attendant on the management of the case during the abscess stage.

All authorities on rectal surgery emphasize the gravity and importance of the condition under consideration, and its liability to result in fistula when improperly managed. VanBuren has cited a number of cases in confirmation of this fact, one of which I take the liberty to quote :

> A very busily-employed executive officer in a heavy financial institution, sitting all day and living a little too well at home, was a victim of one of the most extensive abscesses of this sort I ever saw. His health had always been excellent and he was in the prime of life, but the abscess which formed in midwinter proved to be the cause of his death. Its formation and progress were insidious and slow, and its opening was long deferred and then not made sufficiently free ; and when I saw him in consultation there was an extensive cavity extending nearly around the circumference of the bowel, and he was suffering from hectic fever, which, as you know, is good evidence that the vital powers of a patient are unequal to the repair of his disease. We succeeded, however, in improving his condition so that he could have undertaken a sea voyage with a good prospect of ultimate cure ; but he refused to abandon his business and died exhausted during the subsequent midsummer heats.

Allingham, who has had so large an experience at St. Marks' Hospital, in London, states that out of 190 persons suffering from ischio-rectal abscess, 151 had terminated in fistula.

Curling, Ball, and others, all unite in saying that unless properly and energetically treated, the form of abscess under consideration is very liable to be followed by fistula.

In speaking of the treatment of these abscesses, a celebrated author says :

> The surgeon should make an early and free opening with the knife through the integument and follow it with his finger, so as to secure a direct and sufficient outlet, not only for pus but for sloughy *débris*.

This affords the only assurance of safety. When it is neglected, extensive surface ulceration and sloughing are liable to follow, with an amount of destruction of pelvic tissue around the lower end of the gut, which is often irreparable, and where the patient does recover he is liable to permanent disability.

A more severe form of abscess, so far as prognosis is concerned, than the latter, is what is known as abscess of the superior pelvi-rectal space, but, fortunately, this variety is more rare than ischio-rectal abscess. When we consider for a moment the anatomical bearings of the pelvi-rectal space, it can be readily understood that an abscess in this locality is a serious affair, indeed. We can see from a review of our anatomy why it is that an abscess in this locality assumes sometimes such vast proportions, burrowing into the connective tissue of the illiac fossa or anywhere else in the pelvic basin. These abscesses have been known to discharge into the vagina, bladder and rectum, and they may point in the loin, groin or gluteal region. They may cause urinary retention and fecal obstruction. The etiology of these abscesses comprise all the known causes of ischio-rectal abscess together with parturition (injury by pressure of the fetal head or blood-poisoning), diseases of the urinary organs, notably gonorrhea, acute prostatitis, urethral rupture with urinary extravasation, and the introduction of foreign bodies. An interesting case was recorded a few years ago by a French writer, where a hairpin had been introduced into the rectum, which subsequently worked its way into the sigmoid flexure, and after two years formed an abscess which opened first in the lumbar region, then over the trochanter major, and finally in Scarpa's triangle. This case was supposed to be one of hip-joint disease, but an operation revealed the error in diagnosis. On account of the obscurity of the symptoms, the diagnosis of this form of abscess is sometimes very difficult. Under this head Kelsey says: "The diagnosis of deep pelvic abscess will not often be made before the tumor becomes apparent or the pus has discharged itself. After a few days of sickness a hard, painful tumor appears in the lower part of the abdomen, or a free discharge of pus takes place from the rectum or bladder, and the cause of the previous symptoms becomes apparent. The rupture may occur into the peritoneal cavity, and a general peritonitis result, though in men the tendency is rather downward along the rectum to the perineum. Careful digital examination of the rectum will often render an otherwise obscure case perfectly plain. Even when the diagnosis of pelvic abscess has been made, it may be impossible for a time to decide upon its origin, for psoas abscess, abscess from hip disease, perityphlitis, periproctitis, perinephritis, and inflammation in the connective tissue of the illiac fossa, may each cause a collection of pus in the pelvis. After pus has been obtained, should the origin not be plain, a microscopic examination for bone tissue may make it so."

The prognosis of this form of abscess is necessarily somewhat grave. Statistics prove that about twenty per cent. of the cases terminate fatally. Where the patients do not die, they are frequently left in a crippled condition, on account of the extensive burrowing of the abscess, resulting sometimes in permanent fistula and sometimes in rectal stricture, caused by cicatricial contraction. Ségoud, who has collected some important statistics regarding the termination of these cases, states that out of 112 cases,

> Thirty-five perforated the urethra, and seventy-seven, other parts, generally the rectum, but occasionally the perineum, the ischio-rectal fossa, and the obturator foramen. Twenty per cent. are fatal, and many leave fistulous communications with the urethra and rectum which are never cured.

The treatment of these cases consists of free evacuation of the pent-up matter, together with the use of antiseptic washes and perfectly free drainage. The patient should be built up by a nourishing, generous diet, cod liver oil, iron, hypophosphites, etc. A change of climate, and, in some cases, a sea voyage, may be followed by beneficial results.

271 FRANKLIN STREET.

---

POTASSIUM IODIDE IN THE DIAGNOSIS OF PHTHISIS.—An observation, which a few years ago was made by Sticker, is being confirmed recently by the same author, in regard to a peculiar action of potassium iodide in doubtful cases of phthisis. It appears that, in instances of this disease, when no rale or blowing murmur is detected at the apex of the lung, the seat of the lesion, small doses of the drug gave, in a few days, rise to the appearance of wanting signs. The medicine seemed to stimulate secretion, especially in the neighborhood of dead tissues, thus producing rales. In four cases of suspected tuberculosis, where negative results were obtained from physical examination of the lungs, the ingestion of the potassium salt, for purposes of diagnosis, produced distinct signs of localized reaction.—*Columbus Medical Journal.*

## Figures and Tables

**Figure f1:**